# Progress in Psychiatry

**Published:** 1903-11-07

**Authors:** 


### PROGRESS IN PSYCHIATRY.

**Multiple and Alternating Personalities in Hypochondriasis.**—Recent clinical investigations in regard to the subject of so-called "double consciousness" have brought to light many facts of medical and medico-legal interest. In the prosecution of these studies hypnotic methods have served to ascertain facts concerning the nature of the mental disorder apparently unascertainable by other means, and hypnotism has, in the hands of skilled physicians, proved beneficial in the treatment of such cases. Dr. Ira van Giesen[1] records the case of a Russian, aged 26 years, and of good family history. In February, 1900, he began to suffer from insomnia, headaches and loss of appetite, and had become despondent. His general health deteriorated, and a local physician on examining him stated that his trouble was largely indigestion, and that there were "lumps" in the bowel. This became the starting-point of a delusion which gradually grew more complex. The patient believed that there were worms in the intestine which "worked upon" these lumps, broke them up into smaller lumps and disseminated the latter throughout the body. A delusional fabric (attempt at explanation) grew up gradually around these morbid ideas. The patient believed that he was rescued from dire distress by three agencies, viz., the spleen, the soul, and the veins, the soul being the scavenger and the spleen the director. "When the attention of the soul was distracted this work (of eliminating the 'lumps') was not well done." He passed into a mood of extreme depression, could only speak readily of the all-pervading subject of his delusion, paid little attention to surrounding objects (apathy) and reacted slowly to external stimuli. No gross motor disturbances were present. There were no hallucinatory or other sensory disturbances, and no tendency to suicide. He could fully realise his environment and his relations to space and time. On putting him into a hypnotic trance, says Dr. Giesen, a metamorphosis occurred, the patient passing from intense depression to a state of great exaltation. Despite this the central delusion persisted and appeared to be far better organised. This clearly pointed to one of two things—that either the state of depression was a secondary condition resulting from the delusion, or that the delusion being secondary in its origin, *i.e.*, secondarily arising on a basis of pre-existing physical and emotional depression, had so gained in strength and so impressed itself in the higher centres of the brain as to be able to stand by itself even after the emotional basis had been withdrawn. The latter alternative seemed the more probable, being more consistent with the known pathogenesis of delusions of this type. In this condition of hypnotic trance he could vividly remember all the events of his waking life. On a later occasion, when hypnotised, the patient passed into a deeper trance in which the mood underwent a change from a state of exaltation and delight (the state proper to the lighter degree of trance alluded to above) to one of grave composure. In this last trance-state he could remember all the experiences of the other trance personality and of the waking state. It was thus rendered clear that three personalities, each with its characteristic and dominant features, existed in the patient at different times. Of the three personalities the melancholic one which characterised his waking life was clearly pathological, since it was acquired as the result of illness—bodily and emotional depression attending gastro-intestinal trouble. This melancholic personality, which was dominant and permanent in his waking state, disappeared under hypnosis, giving place (temporarily) to his normal personality or personalities. But the trance personality itself was divisible into two personalities, the one exalted and over-expressing itself, the other of quiet and composed demeanour. The latter corresponded most closely to the patient's healthy condition—before the period of hypochondria. These alternating personalities, however, were ephemeral, and under treatment (the method of which is detailed below) the two abnormal personalities disappeared. The first to shrink and disappear was the melancholic personality of the patient's waking state. In course of time the other personality—the first or exalted trance personality, also disappeared never to return again. The mood of the second trance personality (which approximated closest to the normal life of the patient) then lost some of its former seriousness and persisted as the dominant one of normal life thereafter. But throughout all these transformations the central delusion which had established itself in the brain during illness remained unshaken. "The great assimilating power of the delusions," says Dr. Giesen, "was wonderful. Various 'suggestions' were given to the patient; although they were designed to break up the nucleus of the delusion they were turned about by the patient and fed into this systematised delusion." (This recalls the analogy of the pathogenesis of paranoia). But other lines of treatment were tried and success followed at last when the whole mechanism of hypnosis was understood and effectively applied. The patient being deeply hypnotised "suggestions" of the following kind were first instilled into him, viz., that the unpleasant thermal changes in the "lumps" were abolished and had vanished. These were only partially successful, and to increase the effect of suggestion the method of emotional stimulation coupled with suggestion was employed. Thus while in a state of deep hypnosis, dreams were suggested to the patient with the object of effecting changes in the central delusion. For example, in one of these dreams his father told him that these lumps would go away. "These dreams impressed him deeply though slowly." When the good dreams had become dominant, the "spleen" and "soul" began to drop out from their place in the drama of his delusion, and then the actual "galvanic current was substituted for the soul with great benefit." "Small spots" were next substituted for the large "lumps." These spots were then gradually confined to certain areas instead of being vaguely disseminated, and eventually they vanished taking away with them the *raison d'être* of the elements of the delusion itself. The patient's physical health now improved concurrently with the disappearance of his delusions, and he was eventually able to resume his original vocation. The state of cure was permanent, and 18 months afterwards the patient was shown at the New York Neurological Society.

[1] Jour. of Nerv. and Ment. Diseases, November 1902.

(*To be continued.*)

